# Electric field induced reversible 180° magnetization switching through tuning of interfacial exchange bias along magnetic easy-axis in multiferroic laminates

**DOI:** 10.1038/srep16480

**Published:** 2015-11-18

**Authors:** Xu Xue, Ziyao Zhou, Bin Peng, Mingmin Zhu, Yijun Zhang, Wei Ren, Tao Ren, Xi Yang, Tianxiang Nan, Nian X. Sun, Ming Liu

**Affiliations:** 1 Electronic Materials Research Laboratory, Key Laboratory of the Ministry of Education & International Center for Dielectric Research, Xi’an Jiaotong University, Xi’an 710049, China; 2 Energy Systems Division, Argonne National Laboratory, Lemont, IL, 60439, USA; 3 Beijing Education Examinations Authority, Beijing 100083, China; 4 School of Information and Electronics, Beijing Institute of Technology, Beijing 100871, China; 5 Electrical and Computer Engineering Department, Northeastern University, Boston, MA 02115, USA; 6 Collaborative Inovation Center of High-End Manufacturing Equipment, Xi’an Jiaotong University, Xi’an, 710049, China

## Abstract

E-field control of interfacial exchange coupling and deterministic switching of magnetization have been demonstrated in two sets of ferromagnetic(FM)/antiferromagnetic(AFM)/ferroelectric(FE) multiferroic heterostructures, including NiFe/NiCoO/glass/PZN-PT (011) and NiFe/FeMn/glass/PZN-PT (011). We designed this experiment to achieve exchange bias tuning along the magnetic easy axis, which is critical for realizing reversible 180° magnetization deterministic switching at zero or small magnetic bias. Strong exchange coupling were established across AFM-FM interfaces, which plays an important role in voltage control of magnetization switching. Through the competition between the E-field induced uniaxial anisotropy in ferromagnetic layer and unidirectional anisotropy in antiferromagnetic layer, the exchange bias was significantly shifted by up to |∆H_ex_|/H_ex_ = 8% in NiFe/FeMn/glass/PZN-PT (011) and 13% in NiFe/NiCoO/glass/PZN-PT (011). In addition, the square shape of the hysteresis loop, as well as a strong shape tunability of |∆H_ex_|/H_c_ = 67.5 ~ 125% in NiFe/FeMn/glass/PZN-PT and 30 ~ 38% in NiFe/NiCoO/glass/PZN-PT were achieved, which lead to a near 180° magnetization switching. Electrical tuning of interfacial exchange coupling in FM/AFM/FE systems paves a new way for realizing magnetoelectric random access memories and other memory technologies.

Multiferroic heterostructures^1^,^2^,^3^,^4^,^5^, consisting of ferromagnetic (FM)/antiferromagnetic (AFM), ferroelectric (FE) and ferroelastic phases, have shown the great ability of electrically manipulating magnetic properties or vice versa. Particularly, voltage control of magnetization will have promising application on information storage, sensors, and RF/microwave tunable devices. Compared to traditional magnetic field tuning devices, voltage controllable electronic/spintronic devices without bulky, slow and current-driven electromagnet are fast, compact and energy efficient^6^,^7^,^8^,^9^,^10^. Moreover, voltage control of magnetization could enable novel memory devices such as magnetoelectric random access memories (MERAMs)^11^,^12^,^13^,^14^,^15^. These MERAMs combine the advantages of FeRAMs (ferroelectric random access memories) and MRAMs (magnetic random access memories), where the magnetization is non-volatile and switchable with a control voltage. Nevertheless, to realize E-field 180° switching of magnetization is still an open challenge in conventional FM/FE multiferroic heterostructures^7^,^8^,^9^,^10^,^11^,^12^,^13^,^14^,^15^. It is very important to develop a novel coupling mechanism for 180° deterministic magnetization switching in multiferroic systems.

The discovery of exchange bias (H_ex_) by Meiklejohn and Bean^16^ in 1956 opened up one of the most important research fields because of its key role in giant magnetoresistive spin value^17^,^18^. The *H*_ex_ arises from the exchange coupled ferromagnetic (FM) and antiferromagnetic (AFM) layers across their interface^19^,^20^,^21^, which results in a shift in the hysteresis loop of FM layer and mainly depends on various factors such as the FM and AFM layer thicknesses^22^,^23^, degree of crystallization of AFM layer^24^, FM/AFM interface structure^25^, and magnetic annealing treatment^26^. Recently, researchers have devoted their efforts to explore E-field control of exchange bias due to its potential application in MERAM devices and significant progress has been achieved in this field^11^,^15^,^27^,^28^,^29^. Laukhin *et al.* revealed that magnetic bias can be tuned with E-fields in FM/YMnO_3_ (AFM/FE) multiferroic heterostructure^27^. Later, Wu *et al.* discovered that in La_0.7_Sr_0.3_MnO_3_ (FM)/BiFeO_3_ (FE and AFM) system, exchange bias and coercive field can be manipulated by E-field^28^. By using Co/Pd multilayers as ferromagnetic layer and Cr_2_O_3_ as antiferromagnetic layer, He *et al.* obtained E-field controllable non-volatile exchange bias in that heterostructure^15^. Further, based on the progresses in FM/FE multiferroic heterostructures, Liu *et al.* created a novel AFM/FM/FE multiferroic heterostructure of FeMn/Ni_80_Fe_20_/FeGaB/PZN-PT and observed a significant E-field control of exchange bias field which is up to |ΔH_ex_|/H_ex_ = 218%^11^. Some follow up work has been done by Giang *et al.* in IrMn/Co/PZT multilayers, where magnetization reversal was obtained by E-field control of exchange bias^29^. The key point of constructing AFM/FM/FE structure is to achieve 180° magnetic switching.

Although large exchange bias tunability was achieved in previous work of multiferroic multilayer structure FeMn/Ni_80_Fe_20_/FeGaB/PZN-PT (>200%, ref. 11), challenges still remain in E-field control of exchange bias and switching of magnetization. For instance, along the magnetic easy-axis, the electric field induced exchange bias change is zero and that will put a limitation on to realize a reversible near 180° deterministic magnetization switching at zero or small magnetic bias field, which is critical for E-field tuning memory devices. To fulfill this propose, a square shaped hysteresis loop and large exchange bias along the easy axis are required. The AFM layer with high crystallinity exhibits strong coupling to the FM layer, which further improves the interfacial coupling with the neighboring NiFe layer, thereby enhances the exchange bias field^26^.

In this work, we designed two sets of FM/AFM/FE multiferroic heterostructures, Ni_80_Fe_20_ (NiFe)/Ni_0.5_Co_0.5_O (NiCoO)/glass/PZN-PT(011) and Ni_80_Fe_20_ (NiFe)/Fe_50_Mn_50_ (FeMn)/glass/PZN-PT(011), to obtain E-field control of exchange bias along magnetic easy axis. The E-field induced unidirectional anisotropy associated with the unidirectional interfacial exchange coupling between NiFe and NiCoO (or FeMn) is tunable by adjusting the in-plane magnetic easy axis of NiFe along either [100] or [0
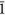
1] of PZN-PT. Through competition between the E-field induced uniaxial anisotropy and unidirectional anisotropy, large E-field induced tunable exchange bias field of up to |∆H_ex_|/H_ex_ = 13% (or 8%) was demonstrated in the NiFe/NiCoO (Or FeMn)/glass/PZN-PT multilayer films. Most importantly, the antiferromagnetic anisotropy could be changed by the E-field induced PZN-PT strain. Therefore, we observed a significant shift of the magnetic hysteresis loop along the magnetic easy axis, which is the key to realize a complete reverse of magnetization.

## Results

NiFe is a typical soft magnetic material with high squareness that fulfills our purpose. FeMn and NiCoO are ideal antiferromagnetic materials that can create high AFM/FM interaction with NiFe. Also, The (111) crystal orientation of FeMn will create (111) crystal orientation of NiFe that enhances the squareness of magnetic hysteresis loops. NiFe (30 nm)/NiCoO (50 nm) and NiFe (30 nm)/FeMn (30 nm) multilayer films were grown on glass substrates by magnetron sputtering with a base pressure below 1 × 10^−7^ Torr at room temperature. It is worth to emphasize that we are not trying to achieve larger exchange bias tunability than that of ref. 6but to realize exchange bias tuning through E-fields along the magnetic easy axis at the first time in this work. Therefore, we grew AFM/FM bilayer thin films onto smooth glass substrates to achieve a well-defined interfacial exchange coupling system with a square shape of hysteresis loop and large exchange bias. Although *in situ* growth of AFM/FM bilayer structure onto PZN-PT substrates may bring larger exchange bias tunability (as ref. 11), the continuity of exchange interaction may be broken by kinks at domain boundary in multi-domain structural PZN-PT (011) single crystal during the application of E-field. During the deposition, an external magnetic field of 200 Oe was applied to form the magnetic easy axis of NiFe film. As we expected, large exchange bias field was established as 15.4 Oe in NiFe/NiCoO/glass/PZN-PT multilayers and 125 Oe in NiFe/FeMn/glass/PZN-PT multilayers due to smooth surface condition of glass, respectively, and that will give a solid foundation of realizing exchange bias tuning through E-field along easy axis.

(011)-cut ferroelectric PZN-PT single crystal exhibits anisotropic in-plane piezoelectric coefficients with a negative d_31_ of −3000 pC N^−1^ along [100] and a positive d_32_ of 1000 pC N^−1^ along [0
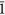
1]^30^. These anisotropic in-plane piezoelectric coefficients of PZN-PT produce a compressive stress along [100] direction and tensile stress along [0
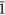
1] direction, thus providing an opportunity for achieving a large change of in-plane uniaxial anisotropy in its bonded NiFe layer. We epoxy-glued the NiFe/NiCoO/glass or NiFe/FeMn/glass multilayer structures onto PZN-PT substrate with magnetic easy axis along [100] or [0
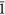
1] PZN-PT crystal directions. An E-field applied across the thickness direction of the PZN-PT (011) substrate leads to an E-field-induced effective uniaxial magnetic anisotropy field along the [100] direction of PZN-PT through strain mediated magnetoelectric (ME) coupling.

**Figure 1:**
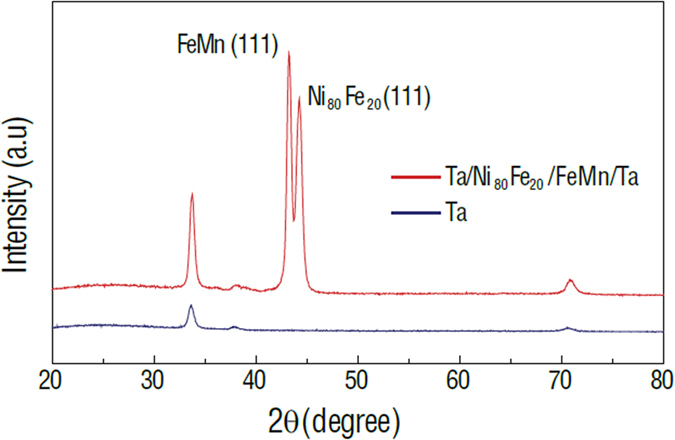
X-ray diffraction patterns of the Ta/NiFe/FeMn/Ta/glass multilayer films. FeMn (111) and NiFe (111) peaks were shown in the figure.

**Figure 2:**
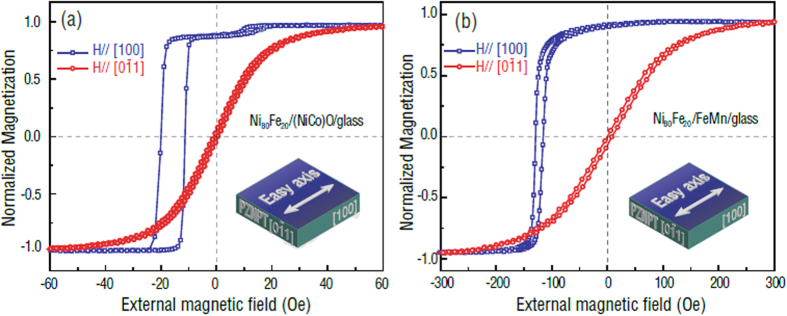
Magnetic hysteresis loops. (**a**) NiFe/NiCoO/glass/PZN-PT multilayer films along the magnetic easy and hard axis. (**b**) NiFe/FeMn/glass/PZN-PT multilayer films along the magnetic easy and hard axis.

Figure 1 shows the X-ray diffraction patterns of the Ta/NiFe/FeMn/Ta/glass multilayer films. Pure FeMn and NiFe phases were obtained and confirmed by X-ray diffraction, as shown in Fig. 1, exhibiting both (111) preferred orientation. The FeMn film was deposited as the antiferromagnetic (AFM) layer and the NiFe film served as the ferromagnetic (FM) layer, thus an interfacial exchange coupling between the two materials would be created. Figure 2 shows the magnetic hysteresis loops of the NiFe/NiCoO/glass/PZN-PT and NiFe/FeMn/glass/PZN-PT multilayer films, where the magnetic easy axis is along the [100] direction of PZN-PT. As shown in Fig. 2(a), the magnetic hysteresis loop of NiFe/NiCoO/glass/PZN-PT is square when the external magnetic field is applied parallel to the magnetic easy axis [100], accompanied with an exchange bias (H_ex_) of 16 Oe and a coercive field (H_c_) of 5 Oe. When the external magnetic field is applied along the magnetic hard axis direction (i.e., [0
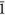
1] direction of PZN-PT), the magnetic hysteresis loop becomes tilted, accompanied with a relatively slow change during magnetization reversal and a negligible H_ex_. That is because the exchange bias production term (S_FM_∙S_AFM_) goes to zero when the external magnetic field is perpendicular to the unidirectional anisotropy of the AF. Similar phenomenon can also be observed in the NiFe/FeMn/glass/PZN-PT multilayer films, as shown in Fig. 2(b), a huge H_ex_ up to 125 Oe with a coercivity field (H_c_) of 8 Oe is observed in NiFe/FeMn/glass/PZN-PT when the external magnetic field is applied along the magnetic easy axis [100], and the magnetization process also becomes harder as the external magnetic field is applied along the [0
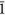
1] direction of PZN-PT. The large H_ex_ in NiFe/FeMn/glass/PZN-PT compared with that in NiFe/NiCoO/glass/PZN-PT indicates a stronger interfacial exchange coupling between NiFe and FeMn layers in comparison to the case of NiFe and NiCoO.

There are minor hysteresis loops biased to the opposite direction of the major loops, Fig. 2(a). The major loop and minor loop show the same strength of exchange bias field. This may be attributed to a thermal process during which an electrode was soldered on the edge of the film in the presence of an external magnetic field. This may reverse magnetic exchange bias in a small region. Since the major loop represents the NiFe/FeCoO/PMN-PT exchange bias behavior. E-field tuning of exchange bias has been demonstrated by observing the major loop shifts. Therefore, we believe the results are sufficient to imply the nature of strain tuning of exchange bias in this major loop. In addition, the amplitude of minor loop is quite small, indicating the inversed exchange bias coupling takes place in a small region over entire film surface, which can be neglected.

**Figure 3:**
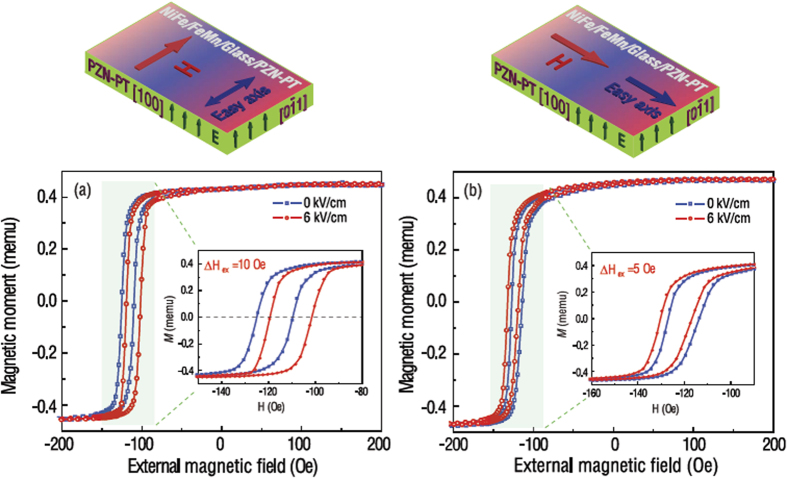
E-field dependence of exchange bias of NiFe/FeMn/glass/PZN-PT multilayers. Magnetic easy axis is along either [0
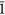
1] (**a**) or [100] (**b**) of PZN-PT. The exchange bias tunability |∆H_ex_|/H_c_ is 125% (along [0
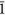
1]) and 67.5% (along [100]).

Figure 3 presents the E-field dependence of exchange bias of the NiFe/FeMn/glass/PZN-PT multiferroic heterostructure for magnetic easy axis along either [0
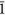
1] (d_32_) or [100] (d_31_) crystal orientations. The (011)-cut PZN-PT single-crystal slab has anisotropic in-plane piezoelectric coefficients of d_31_ and d_32_, which could generate in-plane compressive stress along [100] and tensile stress along [0
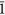
1] while applying an E-field parallel to the thickness direction, i.e. [011] (d_33_) direction of the PZN-PT. This E-field leads to an in-plane deformation of the PZN-PT slab, resulting in contraction and extension along the [100] and [0
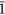
1] directions, respectively. Therefore, the magnetization change in the NiFe layer takes place due to the inverse magnetoelastic effect. In this way, the E-field tuning of magnetization can be realized in the NiFe/FeMn/glass/PZN-PT multiferroic heterostructure through the strain/stress mediated magnetoelastic coupling between NiFe and PZN-PT and E-field tuning of the exchange bias can be also obtained by FM/AFM coupling between NiFe and FeMn. The NiFe/FeMn/glass/PZN-PT multilayer films display an opposite magnetization process when it is magnetized along the two orthogonal in-plane axes, which is reflected by the opposite trend of the E-field dependence of magnetic hysteresis loops and exchange bias shifts. As shown in Fig. 3, an E-field induced reduction of H_ex_ by 10 Oe and enhancement by 5 Oe is achieved when the external magnetic field is applied parallel to the magnetic easy axis [0
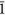
1] and [100], respectively. To switch the magnetization with lower E-field, large change of exchange bias |∆H_ex_| and small coercive field H_c_ are required^11^. In that case, the magnetization can be switched back and forth at given magnetic bias field easily. In order to evaluate the capability of achieving 180° deterministic magnetization switching, we defined the tunability of exchange bias as: |∆H_ex_|/H_c_. Large exchange bias tunability of NiFe/FeMn/glass/PZN-PT about 125% and 67.5% along magnetic easy axis [0
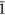
1] and [100], respectively, were obtained in our experiment.

Notice that the NiFe serves as the FM layer and its magnetostriction coefficient *λ* is negative^31^, and the E-field induced effective uniaxial magnetic field can be expressed as equation (1) 32:


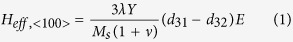


along [100] orientation of the PZN-PT, where *Y* is the Young’s Modulus, *ν* is Poisson’s ratio, *M*_s_ is the saturation magnetization of NiFe. Consequently, when the external magnetic field is applied along the magnetic easy axis [0
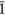
1], as shown in Fig. 3(a), E-field-induced effective uniaxial magnetic anisotropy field H_eff_ in NiFe film along the magnetic hard axis direction could block the magnetization process of the NiFe/FeMn/glass/PZN-PT, resulting in a reduction of exchange bias. On the other hand, as shown in Fig. 3(b), the E-field induced H_eff_ along the magnetic easy axis [100] could facilitate the magnetization process of the NiFe/FeMn/glass/PZN-PT multiferroic heterostructure, which was accompanied with an enhancement of exchange bias and an opposite trend of magnetic hysteresis loop shift.

**Figure 4:**
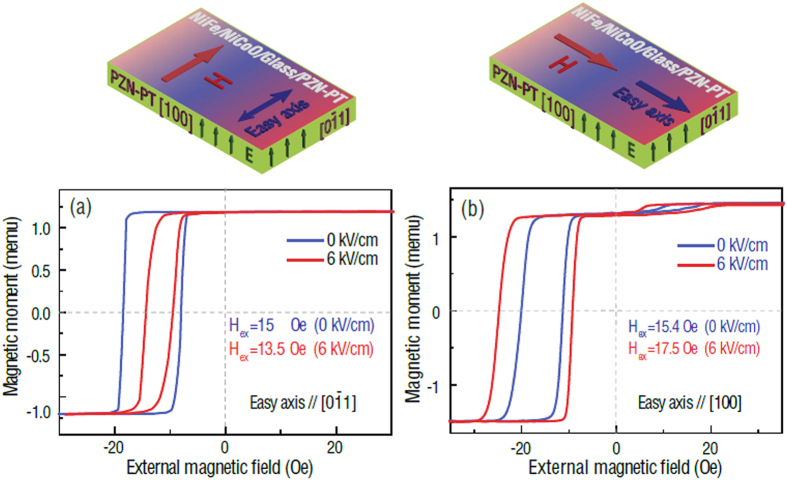
E-field dependence of exchange bias of NiFe/NiCoO/glass/PZN-PT multilayers. Magnetic easy axis is along either [0
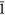
1] (**a**) or [100] (**b**) of PZN-PT. The exchange bias tunability |∆H_ex_|/H_c_ is 30% (along [0
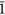
1]) and 38% (along [100]).

Figure 4 presents the E-field dependence of exchange bias of the NiFe/NiCoO/glass/PZN-PT multilayer films for magnetic easy axis either along [0
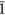
1] or [100] of PZN-PT. The E-field induced exchange bias and coercivity change in NiFe/NiCoO/glass/PZN-PT is more significant compared with that in NiFe/FeMn/glass/PZN-PT as shown in Fig. 3. For the sample with the external magnetic field parallel to the magnetic easy axis [0
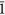
1], H_ex_ was determined to be 15 Oe at zero E-field. Due to the E-field-induced effective uniaxial magnetic anisotropy field which are perpendicular to the external magnetic field, an exchange bias (13.5 Oe) reduction and a notable decrease in coercivity is observed. In contrast, for the sample with the external magnetic field parallel to the magnetic easy axis [100], the E-field-induced effective uniaxial magnetic anisotropy field and the external magnetic field are along the same direction. A much more pronounced E-field tuning of exchange bias was observed, showing a significant enhancement of exchange bias from 15.4 Oe to 17.5 Oe (Fig. 4(b)) while an E-field of 6 kV/cm was applied. Meanwhile, the E-field also produced a notable enhancement in coercivity, indicating that the H_eff_ lifts up the energy barrier during the propagation of domain walls of the ferromagnetic NiFe layer associated with the increased density of pinning sites. Applying an electric field would make magnetization switching process harder or easier with significant exchange bias and coercive field variations for the NiFe/NiCoO/glass/PZN-PT multilayer films by adjusting the external magnetic field along the magnetic easy axis [0
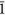
1] or [100]. Through competition between the E-field induced uniaxial anisotropy and unidirectional anisotropy associated with the interfacial exchange coupling, large E-field induced exchange bias tunability of |∆H_ex_|/H_c_ = 30% and 38%, corresponding to external magnetic field along the magnetic easy axis [0
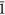
1] or [100], are demonstrated in NiFe/NiCoO/glass/PZN-PT.

The exchange bias field is around 20 Oe in NiFe/NiCoO multilayer which is much smaller than that of NiFe/FeMn system (~100 Oe), indicating a weak pinning effect in NiFe/NiCoO heterostructure. The exchange bias pinning strength of NiFe layer and NiCoO layer is relatively weaker than that of NiFe/FeMn bilayer structure. Therefore, by applying a mechanical strain on these systems, a strong effect would be expected in a weakly pinned exchange bias system of NiFe/NiCoO. The strain/stress induced effect is relative larger when the exchange bias effect is small. The strain/stress change generated by PZN-PT can easily drive the NiFe magnetic moment in NiFe/NiCoO system, instead of NiFe/FeMn system.

**Figure 5:**
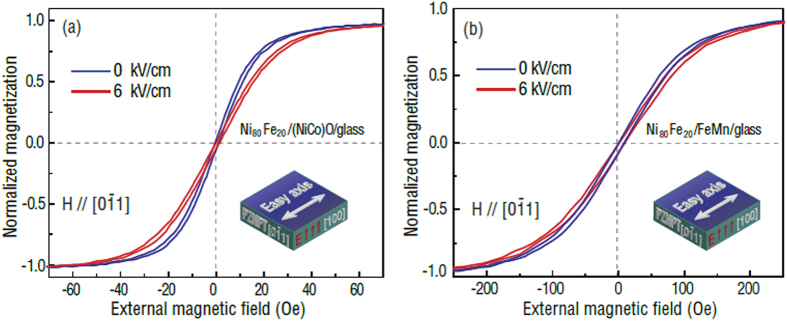
E-field tuning of magnetic hysteresis loops. (**a**) NiFe/NiCoO/glass/PZN-PT multilayer films. (**b**) NiFe/FeMn/glass/PZN-PT multilayer films.

Figure 5 presents the E-field tuning of magnetic hysteresis loops of NiFe/NiCoO/glass/PZN-PT and NiFe/FeMn/glass/PZN-PT multilayer films with the external magnetic field parallel to the [0
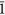
1] direction of PZN-PT while the magnetic easy axis is along [100] direction. As shown in Fig. 5, the tilted magnetic hysteresis loops were reasonably due to the fact that the external magnetic field were applied perpendicular to the magnetic easy axis, and no noticeable exchange bias could be observed in both multilayer films. The in-plane magnetization process of the multiferroic composite displays E-field dependent characteristics while applying an electric field (6 kV/cm) across the thickness direction of PZN-PT. The E-field induced effective uniaxial magnetic anisotropy field along the [100] direction of PZN-PT, which was perpendicular to the external magnetic field direction, would hinder the magnetization process of the multilayer films. Consequently, the more tilted magnetic hysteresis loops accompanied with a negligible change in the exchange bias field were observed in Fig. 5. However, the E-field dependence of the magnetic hysteresis loops in NiFe/NiCoO/glass/PZN-PT is more notable compared with that in NiFe/FeMn/glass/PZN-PT. This is attributed to the stronger interfacial exchange coupling strength of FeMn/NiFe (AFM/FM) than that of NiCoO/NiFe, which is consistent with the different magnitude of exchange bias fields presented in Fig. 2. So in NiFe/FeMn/glass/PZN-PT system, the NiFe moment is much harder to be tuned by E-field induced H_eff_ because it is strongly pinned by more interfacial spins of FeMn^26^. Therefore, in a real memory device, especially for the FM/AFM/FE configuration, if the FM layer is fixed, choosing a weak AFM pinning layer would be beneficial to achieve E-field control of magnetization.

The hysteresis loops shift by E-field in Fig. 5 indicates that the films, as well as glass, are subjected to a compressive strain with applying an electric field. Generally, the epoxy-glued multiferroics heterostructures^33^ have been widely used in tunable microwave devices as seen^34^,^35^,^36^. A strong mechanical strain transfer between the two epoxy-glued ferromagnetic and ferroelectric lays has been demonstrated in these reports. Therefore, in our case, a deformation definitely can be transferred to the films from PZN-PT. The reason why we use 6 kV/cm as typical applied E-field value is that there exists a phase transition in PZN-PT generates large strain/stress change. A corresponding large ME effect was systematically studied in our previous FeGaB/PZN-PT work^12^: as we apply E-field larger than 6 kV/cm, the ME effect change is very small. In conclusion, 6 kV/cm is an ideal E-field value for this experiment^12^.

**Figure 6:**
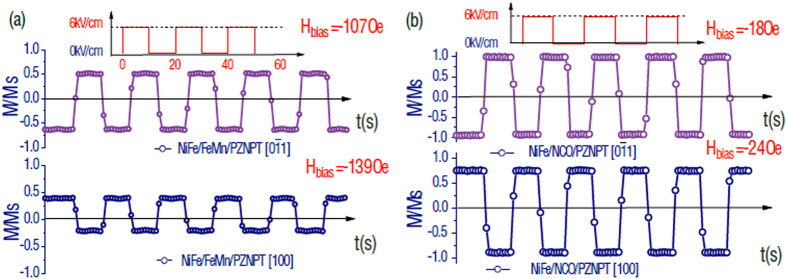
E-field deterministic switching of magnetization through E-field modulating exchange bias in FM/AFM/FE multiferroics. (**a**) Magnetization switching by E-field in NiFe/FeMn/PZN-PT multiferroic heterostructure at bias magnetic field of 107 Oe and 139 Oe, magnetic easy axis is along PZN-PT [0
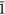
1] and [100], respectively; (**b**) Magnetization switching by E-field in NiFe/NiCoO/PZN-PT multiferroic heterostructure at bias magnetic field of 18 Oe and 24 Oe, magnetic easy axis is along PZN-PT [0
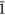
1] and [100], respectively.

The significant E-field dependence of exchange bias along the easy axis in AFM/FM/FE heterostructures provides great opportunities for realizing electrically deterministic magnetization switching in NiFe films. Figure 6 shows the magnetization switching in NiFe films at external magnetic bias fields along PZN-PT [0
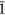
1] and [100] directions. A square wave of electric field with the period of 20 s was applied across PZN-PT substrates. By manipulating the magnetic hysteresis loops along the easy axis, the magnetic moment can be reversed at a given external magnetic bias field^11^. For NiFe/FeMn/PZN-PT multiferroic heterostructures, external magnetic bias fields of 107 Oe and 139 Oe (opposite to exchange bias direction) were applied along the PZN-PT crystal directions of [0
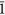
1] and [100]. The magnetization can be switched with applying an electric field (from 0.5 M_S_ to −0.6 M_S_ along PZN-PT [0
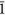
1]; from 0.4 M_S_ to −0.2 M_S_ along PZN-PT [100]), see Fig. 6(a). In contrast, the magnetization switching angles are not nearly 180°. In contrast, within NiFe/NiCoO/PZN-PT multiferroic heterostructures, bias magnetic fields of 18 Oe and 24 Oe (opposite to the exchange bias direction) were applied along PZN-PT crystal directions of [0
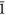
1] and [100]. Figure 6(b) shows that the switching magnetization is very close to the saturation magnetization (from ~M_S_ to −0.95 M_S_ along PZN-PT [0
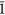
1]; from 0.76 M_S_ to −0.9 M_S_ along PZN-PT [100]) and that implies near 180° magnetization reversals were achieved in NiFe/NCO/PZN-PT multiferroic heterostructures, especially, along PZN-PT [0
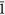
1] crystal direction. The magnetization orientation is along applied magnetic field direction at ~Ms, in contrast, magnetization orientation is opposite to applied magnetic field direction at ~−Ms. The magnetization switching from ~Ms to ~−Ms implies a 180° magnetization reversals from magnetic hysteresis loop measurements. It is worth to mention that the tunability metric |∆H_ex_|/H_c_ is not the only factor that affects the tunability of magnetization. The squareness of hysteresis loops is also important factor. From Figs 3 and 4, it is clear that the hysteresis loops of NiFe/FeMn are more tilted than that of NiFe/NiCoO. Therefore, the magnetization along certain bias field of NiFe/FeMn is not fully saturated. As a result, it is difficult to switch the magnetization 180° in NiFe/FeMn system. In future, we will put effort on to achieve high squareness magnetic hysteresis loops in NiFe/FeMn system.

## Discussion

To further understand the interfacial exchange coupling mechanism, the free energy density of a modified Stoner–Wohlfarth type magnetic layer in AFM/FM/FE multiferroic heterostructure can be written as Equation (2) and (3) 11:


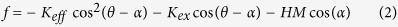



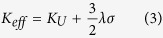


Where *K*_*eff*_ is the effective uniaxial anisotropy constant that takes into account the induced uniaxial anisotropy constant *K*_u_ and the E-field induced magnetic anisotropy constant 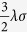; *K*_*ex*_ is the anisotropy constant associated with the unidirectional interfacial exchange coupling between FM and AFM layers; *θ* represents the direction of the easy axis of the FM that is programmed by the 200 Oe field applied during deposition; and *α* is the angle between the applied magnetic field and the magnetization. In Figs 3 and 4, among different AFM/FM/FE multiferroic heterostructures, we studied the cases that the directions of exchange bias and effective anisotropy were parallel or perpendicular to each other. According to the theoretical calculation and experimental data^6^, in both cases where exchange bias direction is parallel or perpendicular to effective anisotropy direction, if *K*_*ex*_ is constant there is no E-field induced exchange bias shift along magnetic easy axis in AFM/FM/FE heterostructures. Our observation is, nevertheless, opposite to the prediction, in both Figs 3 and 4, there exists significant easy axis exchange bias changes in NiFe/NiCoO/glass/PZN-PT and NiFe/FeMn/glass/PZN-PT. We could make an assumption that the *K*_*ex*_, interfacial exchange coupling anisotropy, depends on E-field induced strain/stress change. However, the mechanism of E-field control of exchange bias along magnetic easy axis still needs further study. One possible reason could be that the magnetic unidirectional anisotropy *K*_*ex*_in the AFM layer is manipulated by E-field and then influences the exchange interaction between FM and AFM layer, thus, changes the exchange bias field along easy axis. In this work, we need a bias field to switch magnetization back to the initial state. In future investigation, we will try different FM/AFM/FE exchange bias system to eliminate the bias field by properly shifting the hysterias loops in a small of range with covering the original point. The essential of electric field control of exchange bias tuning along easy axis will be revealed in our future research.

## Conclusions

In summary, two sets of FM/AFM/FE multiferroic heterostructures, NiFe/NiCoO/glass/PZN-PT and NiFe/FeMn/glass/PZN-PT, have been investigated in the present work. With an external magnetic field applied parallel to the magnetic easy axis [100], an exchange bias field (H_ex_) of 16 and 125 Oe can be achieved in NiFe/NiCoO/glass/PZN-PT and NiFe/FeMn/glass/PZN-PT, respectively. An applied E-field would make the magnetization switching process harder or easier with significant coercive field variations for the NiFe/NiCoO/glass/PZN-PT multilayer films by adjusting the external magnetic field either along the magnetic easy axis [0
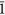
1] or [100]. Through competition between the E-field induced uniaxial anisotropy and unidirectional anisotropy, large E-field induced tunable exchange field of up to |∆H_ex_|/H_ex_ = 13% is demonstrated in the NiFe/NiCoO/glass/PZN-PT multilayer films. In addition, the E-field dependence of the magnetic hysteresis loops in NiFe/NiCoO/glass/PZN-PT is negligible and has no compared with that in NiFe/FeMn/glass/PZN-PT due to the weaker pinning effect form the NiCoO layer.

## Methods

Ni_80_Fe_20_ (NiFe)/Ni_0.5_Co_0.5_O (NiCoO)/glass/PZN-PT and Ni_80_Fe_20_ (NiFe)/Fe_50_Mn_50_ (FeMn)/glass/PZN-PT. NiFe (30 nm)/NiCoO (50 nm) and NiFe (30 nm)/FeMn (30 nm) multilayer films were grown on glass substrates by magnetron sputtering with a base pressure below 1 × 10^−7^ Torr at room temperature. Crystallization structures were examined by X-ray diffraction (XRD) methods. All magnetic hysteresis loops were determined by vibration sample meter (VSM) at room temperature because our target E-field controllable devices are operated at room temperature. Electric fields were applied across the thickness direction of ferroelectric PZN-PT substrates during magnetic hysteresis loops measurement. The thickness of PZN-PT substrates is 0.5 mm and the leakage current by applying the electric field in neglectable, which has no affects to this experiment.

## Additional Information

**How to cite this article**: Xue, X. *et al.* Electric field induced reversible 180º magnetization switching through tuning of interfacial exchange bias along magnetic easy-axis in multiferroic laminates. *Sci. Rep.* **5**, 16480; doi: 10.1038/srep16480 (2015).
